# Preclinical model for phenotypic correction of dystrophic epidermolysis bullosa by *in vivo* CRISPR-Cas9 delivery using adenoviral vectors

**DOI:** 10.1016/j.omtm.2022.09.005

**Published:** 2022-09-16

**Authors:** Marta García, Jose Bonafont, Jesús Martínez-Palacios, Rudan Xu, Giandomenico Turchiano, Stina Svensson, Adrian J. Thrasher, Fernando Larcher, Marcela Del Rio, Rubén Hernández-Alcoceba, Marina I. Garín, Ángeles Mencía, Rodolfo Murillas

**Affiliations:** 1 Unidad de Innovación Biomédica, Centro de Investigaciones Energéticas Medioambientales y Tecnológicas (CIEMAT), 28040 Madrid, Spain; 2 Department of Biomedical Engineering, Carlos III University (UC3M), Madrid, Spain; 3 Centro de Investigación Biomédica en Red en Enfermedades Raras (CIBERER), Madrid, Spain; 4 Fundación Instituto de Investigación Sanitaria de la Fundación Jiménez Díaz, Madrid, Spain; 5 Universidad de Navarra, CIMA, Programa de Terapia Génica y Regulación de la Expresión Génica, Pamplona, Spain; 6 Molecular and Cellular Immunology Unit, UCL Great Ormond Street Institute of Child Health, University College London, London, UK; 7 Great Ormond Street Hospital for Children, NHS Foundation Trust, London, UK; 8 Infection, Immunity and Inflammation Research and Teaching Department, Zayed Centre for Research into Rare Disease in Children, Great Ormond Street Institute of Child Health, University College London, London, UK

**Keywords:** gene editing, epidermolysis bullosa, RDEB, adenoviral vector, CRISPR-Cas, *in vivo* gene therapy, genodermatoses, CAST Seq, Humanized mouse model, Preclinical model

## Abstract

Recessive dystrophic epidermolysis bullosa, a devastating skin fragility disease characterized by recurrent skin blistering, scarring, and a high risk of developing squamous cell carcinoma is caused by mutations in *COL7A1*, the gene encoding type VII collagen, which is the major component of the anchoring fibrils that bind the dermis and epidermis. *Ex vivo* correction of *COL7A1* by gene editing in patients’ cells has been achieved before. However, *in vivo* editing approaches are necessary to address the direct treatment of the blistering lesions characteristic of this disease. We have now generated adenoviral vectors for CRISPR-Cas9 delivery to remove exon 80 of *COL7A1*, which contains a highly prevalent frameshift mutation in Spanish patients. For *in vivo* testing, a humanized skin mouse model was used. Efficient viral transduction of skin was observed after excisional wounds generated with a surgical punch on regenerated patient skin grafts were filled with the adenoviral vectors embedded in a fibrin gel. Type VII collagen deposition in the basement membrane zone of the wounded areas treated with the vectors correlated with restoration of dermal-epidermal adhesion, demonstrating that recessive dystrophic epidermolysis bullosa (RDEB) patient skin lesions can be directly treated by CRISPR-Cas9 delivery *in vivo*.

## Graphical abstract

**Figure:**
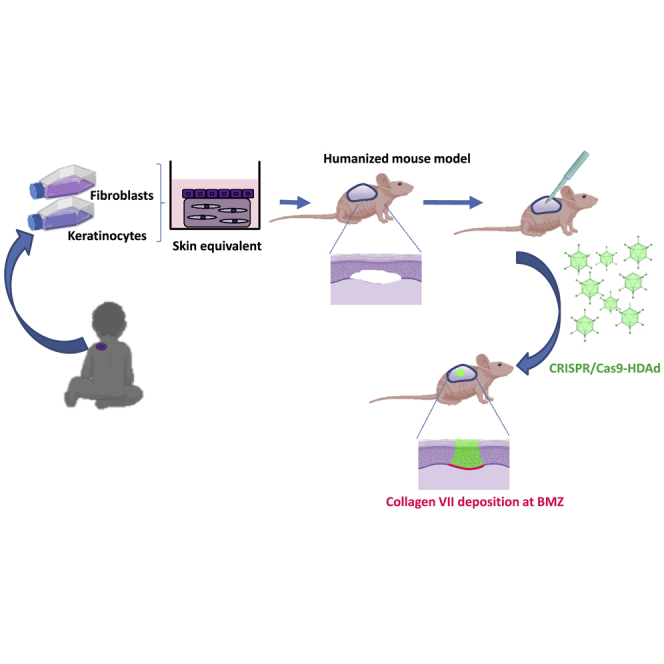

## Teaser

A viral gene therapy approach for the direct treatment of skin lesions in patients with recessive dystrophic epidermolysis bullosa is described. CRISPR-Cas9-mediated deletion of a small fragment of the *COL7A1* gene containing a pathogenic mutation allows restoration of functional collagen VII in a mouse model of humanized patient skin.

## Introduction

Gene-editing-based approaches to modify disease-causing genetic mutations take advantage of cellular mechanisms to repair double-strand breaks (DSBs) introduced by site-specific engineered nucleases, such as CRISPR-Cas9. The most common DNA repair mechanism is non-homologous end joining (NHEJ), which often results in small insertions and deletions at the cleavage site. Protocols based on NHEJ repair are the most likely to be rapidly transferred to the clinic. In fact, several NHEJ-based gene editing strategies are currently in clinical trials, such as T cell engineering for cancer immunotherapy,^1^ recovery of fetal hemoglobin expression,^2^^,^^3^ and correction of Leber congenital amaurosis.^4^ Preclinical studies demonstrate that NHEJ-based gene editing strategies for the deletion of small exons containing pathogenic mutations in genes encoding structural proteins with repetitive domains allow for highly effective correction or improvement of disease phenotypes. These therapeutic exon deletion strategies have been demonstrated for Duchenne muscular dystrophy (DMD)^5^, ^6^, ^7^, ^8^ and recessive dystrophic epidermolysis bullosa (RDEB)^9^, ^10^, ^11^ and could also be tested in clinical trials soon.

Techniques for delivery of the CRISPR system components to modify patient cells *ex vivo* are varied and include polymeric and lipid nanoparticles, electroporation, and viral vectors,^9^^,^^12^, ^13^, ^14^ but *in vivo* delivery remains a challenge.^15^

RDEB is a severe skin fragility disease caused by loss-of-function mutations in *COL7A1*, a gene expressed by keratinocytes and fibroblasts that encodes type VII collagen protein (C7), the main component of the anchoring fibrils required for adhesion between dermis and the epithelial basement membrane. C7 deficiency causes recurrent blistering of the skin and other stratified epithelia, fibrosis, and a high propensity to develop squamous cell carcinomas (SCC) with high metastatic potential.^16^ Currently available therapies are only palliative and marginally effective. Replacement gene therapy is a therapeutic option capable of restoring C7 expression in RDEB cells.^17^^,^^18^ However, gene addition with retroviruses continues to raise biosafety and efficacy concerns.

Our laboratory and others^9^, ^10^, ^11^^,^^19^^,^^20^ have previously developed effective *ex vivo* gene editing protocols for RDEB. We have recently achieved the correction of RDEB in epidermal stem cells from patients by using dual-guide CRISPR-Cas9 delivered as RNPs by electroporation into patient keratinocytes to remove exon 80 of *COL7A1*, the site of a frameshift mutation highly prevalent in the Spanish RDEB population. Exon deletion leads to the expression of an internally truncated protein that is able to restore dermal-epidermal adhesion.

However, although the grafting of *ex vivo* gene-edited skin equivalents is likely to be of great therapeutic utility, it is a complex medical procedure subject to potential complications arising from surgery, and its success depends on graft acceptance, which is quite variable. Complementary *in vivo* treatments for localized lesions could contribute greatly to the clinical care of patients with RDEB. A recent phase I and II clinical trial has demonstrated the feasibility of treating chronic open RDEB wounds with a herpes simplex virus-derived vector for *COL7A1* gene replacement.^21^ This study demonstrates that *in vivo* corrections can be addressed by the use of non-integrating viral vectors, although this gene replacement strategy does not achieve permanent C7 endowment and implies the need for repeated applications. Importantly, the use of an immune-evasive herpes vector potentially allows for repeated treatments. Adenoviral (Ad) vectors that provide high-level transient expression in dividing cells are perfectly suited for *in vivo* gene delivery of CRISPR-Cas9 system elements.^8^^,^^22^^,^^23^ The most widely used Ad vectors are those derived from serotype 5, whose ability to transduce multiple cell types and mediate high levels of exogenous gene expression have been widely demonstrated.^24^^,^^25^ First-generation (FG) vectors retain most of the viral genome, resulting in leaky viral gene expression that can be cytotoxic and trigger immune responses.^26^ Helper-dependent adenoviral (HDAd) vectors are devoid of all viral genes and mediate high-level, long-term transgene expression in the absence of toxicity.^27^

We have now generated Ad vectors to deliver CRISPR-Cas9 components aimed at correcting a mutation that causes RDEB and evaluated their potential for *in vivo* treatment of this pathology by direct application in a humanized skin model of RDEB.

## Results

### FGAd and HDAd vectors for CRISPR-Cas9 expression

To produce the FG vector, a construct for expression of *Staphylococcus aureus* Cas9 (SaCas9) and two guide RNAs was inserted in a pDC315 shuttle plasmid. This construct was cotransfected in HEK293 cells with pGHBGlox, and viral particles were generated after recombination of both plasmids.^26^

**Figure 1:**
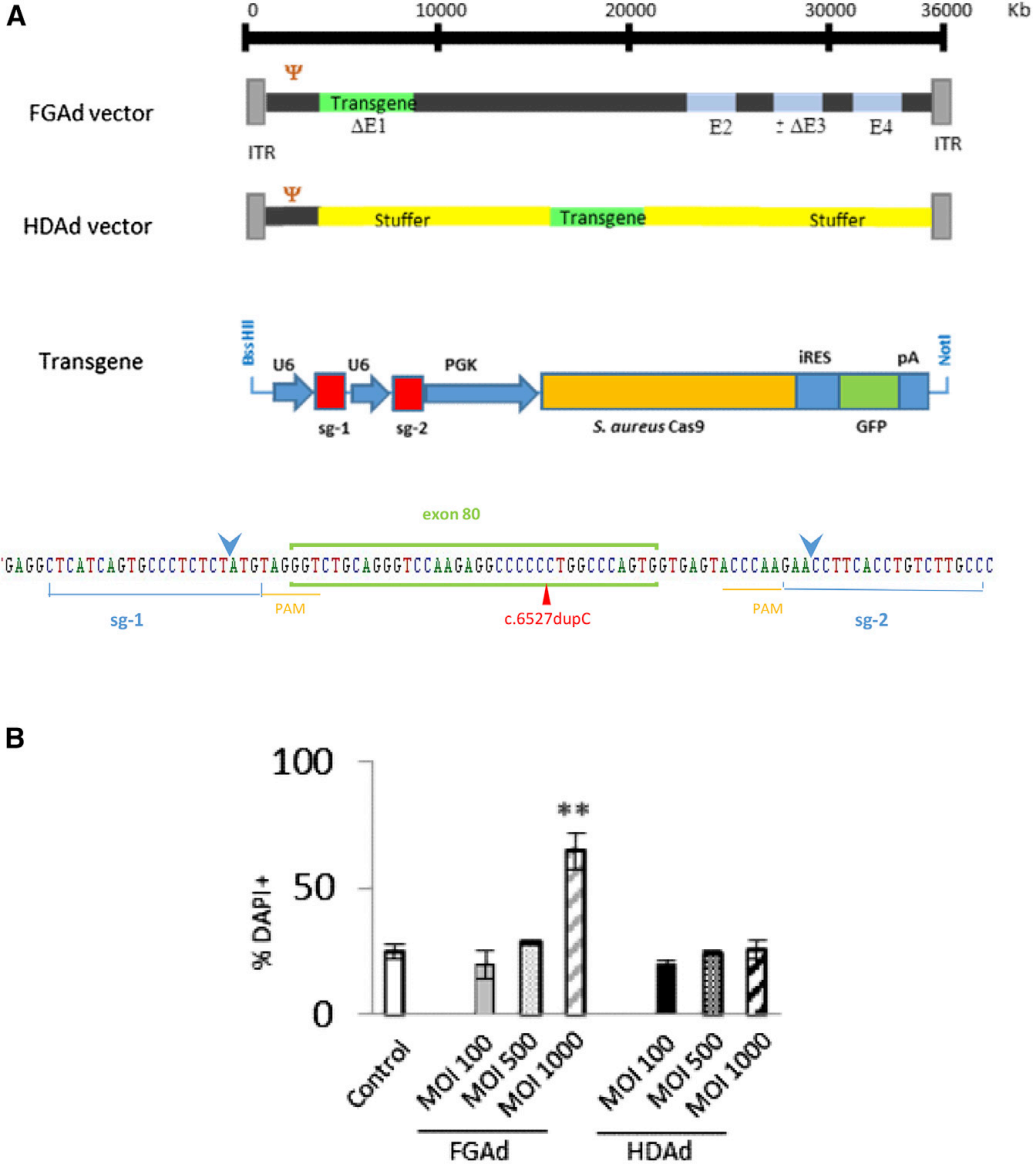
Adenoviral vectors for *COL7A1* exon 80 excision by CRISPR-Cas9 (A) Scheme of transgene for expression of sgRNAs and SaCas9 coupled to GFP in FGAd and HDAd vectors. Bottom panel: position of sgRNAs flanking exon 80 of *COL7A1*. The cut points are marked with blue arrows. c.6527dupC mutation site is shown with a red arrowhead. (B) DAPI permeability assay of patient keratinocytes transduced with growing MOIs with FGAd and HDAd vectors. ∗∗p < 0.01, Student’s t test.

For production of the HDAd vector, the same CRISPR expression construct was cloned into a pC4HSU plasmid that contains Ad5 inverted terminal repeats (ITRs) and encapsidation sequences. Vector particles were produced after transfection of the linearized construct and transduction with helper virus Ad14, which provides all of the viral proteins.^28^ Expression of SaCas9, driven by PGK ubiquitous expression promoter, is coupled to GFP expression by means of an internal ribosomal entry site (IRES) sequence. Guide RNAs that target sequences flanking exon 80 of *COL7A1* are expressed by two cassettes containing the type III U6 promoter (Figure 1A).

Cytotoxicity caused by residual expression of Ad proteins has been described.^29^^,^^30^ To assess the viability of patient keratinocytes after transduction with FGAd and HDAd vectors, cells were transduced with both types of vectors at different multiplicities of infection (MOIs), and DAPI permeability was analyzed by flow cytometry. At the highest MOI, the viability of patient keratinocytes transduced with the FG vector, but not that of those transduced with the HDAd vector, was significantly compromised (Figure 1B and S1). These results confirm the significant cytotoxicity caused by FGAd vectors. Therefore, the HDAd vector was chosen for subsequent *in vivo* studies.

### *COL7A1* exon 80 deletion in cultured patient cells

**Figure 2:**
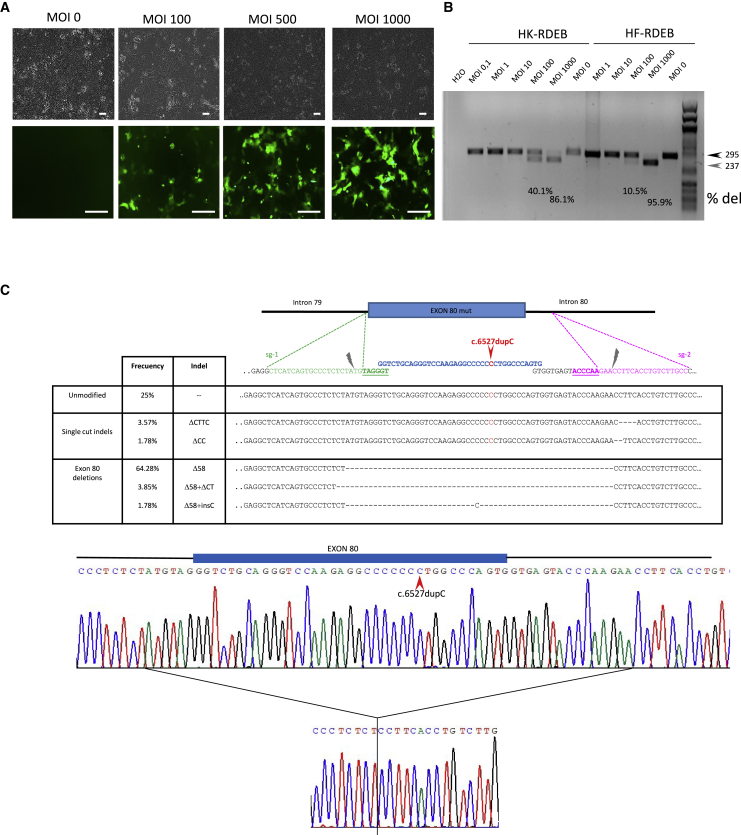
Deletion of *COL7A1* exon 80 in keratinocyte and fibroblast cultures from patients after transduction with HDAd vector for CRISPR-Cas9 expression (A) Transduction efficiency in patient keratinocytes (GFP on bottom panels, phase contrast on top panels) Scale bar: 100 μm. (B) Detection of deletions by PCR analysis after infecting patient cells with growing MOIs. HK, keratinocytes; HF, fibroblasts; RDEB, recessive dystrophic epidermolysis bullosa. The deletion rates assessed by densitometry are shown in each lane. (C) Insertion or deletion (indel) spectrum determined by Sanger sequencing of PCR products corresponding to keratinocytes treated with HDAd vector. A representative chromatogram for Δ58 allele is shown.

**Figure 3:**
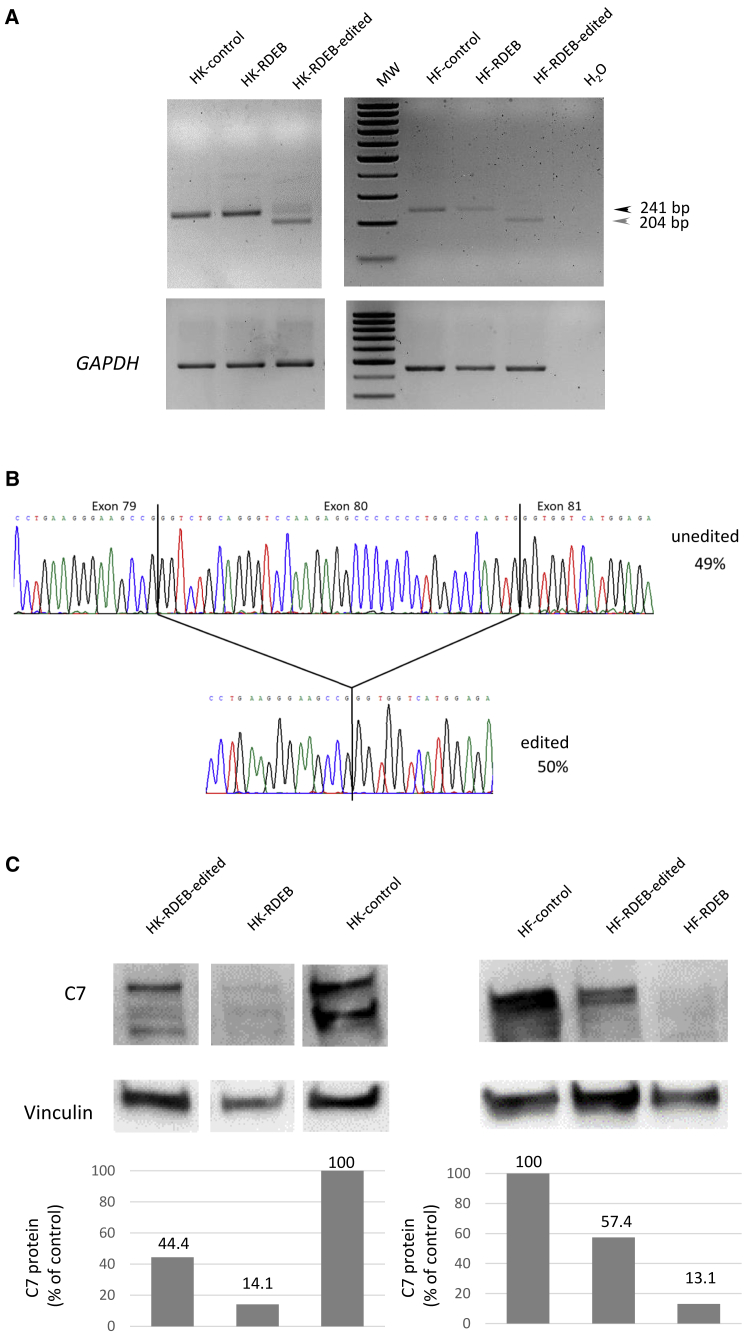
*COL7A1* expression analysis (A) RT-PCR analysis of *COL7A1* transcripts amplified with primers in exons 78–84. Wild-type/c.6527dupC unedited transcripts produced a 240/241 bp band found in all RNA samples. A smaller 204 bp band corresponding to transcripts lacking exon 80 was detected in samples from edited cells. MW, DNA molecular weight marker 100 bp ladder; HK, human keratinocytes; HF; human fibroblasts. *GAPDH* amplification was used as a loading control (bottom panels). (B) Representative sequence chromatograms showing the two different resulting transcripts. Transcript frequencies are shown on the right. (C) Western blot analysis of C7 expression in edited and unedited RDEB and control keratinocytes (left panels) and fibroblasts (right panels). Vinculin expression was used as a loading control (bottom panels). The complete WB membrane for C7 detection is shown in Figure S6.

The ability of the vector to remove the exon 80 of *COL7A1* was first tested in keratinocyte and fibroblast cultures from patients who were homozygous carriers of the c.6527dupC mutation in the *COL7A1* gene. The efficiency of transduction at different MOIs, determined by the expression of GFP (Figures 2A and S2), correlated with the presence of the deletion detected by PCR (Figure 2B). Using an MOI of 1,000, the deletion was found in approximately 85% of *COL7A1* alleles in keratinocytes and 95% in fibroblasts, as determined by the densitometry of the PCR bands. The sequences of the PCR products corresponding to keratinocytes were analyzed by Sanger sequencing of single colonies following bacterial cloning. 70% of sequences corresponded to deletions spanning exon 80, and most of them (65%) were the precise deletion defined by both cutting sites (Figure 2C). To characterize *COL7A1* transcription and C7 protein rescue after gene editing, we performed RT-PCR and western blot (WB) analysis. RT-PCR showed a smaller band consistent with amplification of transcripts lacking exon 80 (Figure 3A). The intensity of the smaller band was proportional to the efficiency of the deletion, as detected by PCR analysis of genomic DNA (Figure 2B). The RT-PCR products corresponding to gene-edited keratinocytes were plasmid cloned (n = 48) and Sanger sequenced. This analysis revealed transcripts containing the proper exon 79-exon 81 junction (50% of transcripts) (Figure 3B) and transcripts containing the exon 80 sequence (49%), while the rest of the transcripts originated from activation of cryptic splicing sequences through the generation of indels. WB analysis performed on cellular extracts demonstrated restored C7 expression in treated cells, both in fibroblasts (right panels) and in keratinocytes (left panels). The uncropped WB is shown in Figure S3. Densitometric analysis of the band corresponding to C7 showed a recovery of C7 synthesis to 44% of the control level in keratinocytes and 57% in fibroblasts (Figure 3C).

The risk of genotoxicity caused by off-target effects of CRISPR is a concern that needs to be addressed for clinical translation of experimental gene editing protocols. Chromosomal aberrations analysis by single targeted linker-mediated PCR sequencing (CAST-seq) is a preclinical assay to identify and quantify chromosomal aberrations derived from on-target and off-target activities of CRISPR.^31^^,^^32^ We have performed CAST-seq analysis of DNA from patient keratinocytes treated with the HDAd vector to detect potential large genomic rearrangements that might result from the dual-guide CRISPR activity. Cells from two patients (P1 and P2), who were homozygous carriers of the c.6527dupC mutation in exon 80 of *COL7A1*, were analyzed. CAST-seq reads surrounding the target site within a 350 bp window showed molecular diversity of deletions (gaps) and inversions (purple bars) caused by the CRISPR system in the target region (Figure S4A). We detected a rare translocation between the on-target site, Chr3:48573840 and ChrX:150501608, likely caused by natural breaks in that locus. This break was found within an intronic region of *MAMLD1* gene. This gene and other genes located within a window of 100 kb from the translocation site are not classified as genes involved in any oncogenic process. The translocation, present in P1 treated cells but not in P2 treated cells, accounted for 4 events out of 150,000 alleles (Figures S4B and S4C). No other translocations were detected, and therefore no off-target sites could be identified with CAST-seq method.

### *In vivo* transduction of wounds in RDEB skin and C7 restoration analysis

To study gene editing correction *in vivo*, we used our skin-humanized mouse model.^33^ We grafted cutaneous equivalents of patient cells, containing both keratinocytes and fibroblasts, onto immunodeficient mice. At 8 weeks post-transplantation, the grafts produced mature skin in which excisional wounds were made with a 2 mm diameter surgical punch, and the fibrin-embedded HDAd vector preparation was deposited in the wound.

**Figure 4:**
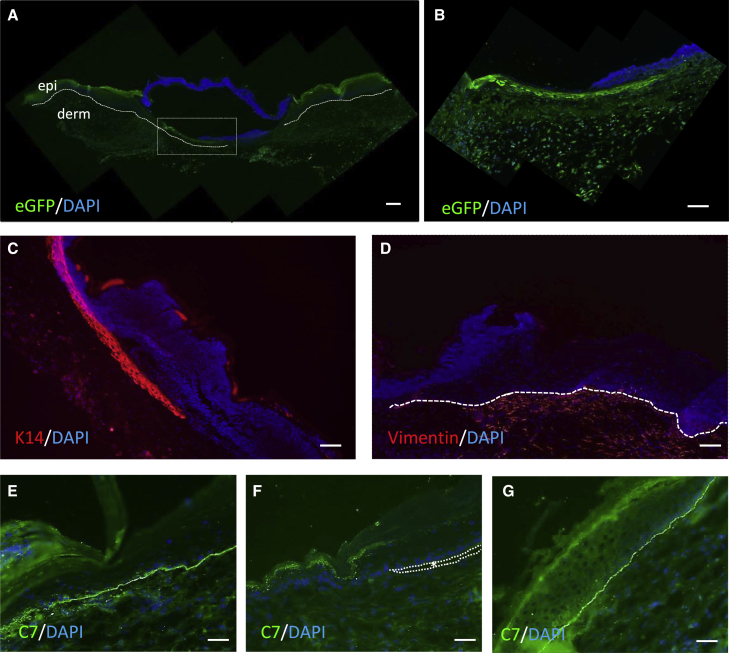
*In vivo* gene-editing-mediated restoration of C7 at the basement membrane of patient skin regenerated onto nude mice (A) Efficient transduction (GFP expression detected by immunofluorescence) of patient skin regenerated on immunodeficient mice after HDAd vector application. Skin samples were analyzed 1 week after treatment. Dotted line underlines the basal epidermal layer and the migratory tongue. Box marks the area magnified in (B). epi, epidermis; derm, dermis. (B) High magnification of the area marked in (A). GFP expression was found in re-epithelialization tongue and the fibroblasts underneath. (C) K14 staining showing the migrating epithelium in the wound. (D) Vimentin staining reveals human dermal fibroblasts migrating to the wound site. (E–G) C7 immunofluorescence detection with monoclonal anti-human C7 antibody in sections of skin regenerated after grafting skin equivalents onto immunodeficient mice. Skin samples were analyzed 2 weeks after vector application. (E) Patient skin, treated with HDAd vector embedded in fibrin. Note continuous C7 deposition at the dermal-epidermal junction. (F) Patient skin, untreated. Asterisks denote blisters. (G) Skin generated from healthy skin cells, positive control. Cell nuclei stained with DAPI (blue). Scale bar: 50 μm.

One week after vector application, wound tissue was removed and processed for histological analysis. Tissue transduction by the Ad vector was visualized by detecting the GFP protein by immunofluorescence with an anti-GFP antibody, showing continuous expression of GFP throughout the epithelial tongue formed in the wound-healing process (Figures 4A and 4B). To show the structure of the migrating epithelium in the wound, we performed anti-K14 staining, which highlights basal keratinocytes (Figure 4C), and anti-vimentin staining, which reveals human dermal fibroblasts migrating to the wound site (Figure 4D). To analyze whether the expression of the *COL7A1* gene had been restored in the transduced cells, we performed immunodetection of C7 in the wound 2 weeks after treatment, sufficient time to detect C7 deposition in the area of the basement membrane zone (BMZ) by the edited keratinocytes and fibroblasts if the treatment had been effective. Continuous C7 deposition along the BMZ underlying the epithelium was found in wounds treated with the vector (Figure 4E), while no staining was found in untreated grafts (Figure 4F). Human skin regenerated from healthy cells was simultaneously stained as a positive control (Figure 4G). Three individual mice carrying a graft derived from patient cells were treated with the vector. Although murine host expression of C7 might be a problem in preclinical models of human C7 recovery in mouse grafts, we used a polyclonal antibody with high reactivity for human C7 and low reactivity for murine C7, which, at the dilution used, only detects human C7.^34^ Furthermore, C7 was only detected in re-epithelialized treated wounds and not in untreated control grafts, in agreement with previous results in which C7 was only detected in grafts from gene-edited patient cells but not in unedited cells.^9^

### Long-term correction of the RDEB phenotype

For genetic correction to be therapeutically useful, it must persist for a long time, which implies that the epidermal stem cells have been edited. To assess whether long-term correction had occurred, tissues were taken from the wounded areas 8 weeks after vector treatment, the time required for two complete epidermal turnover cycles to occur.^35^

**Figure 5:**
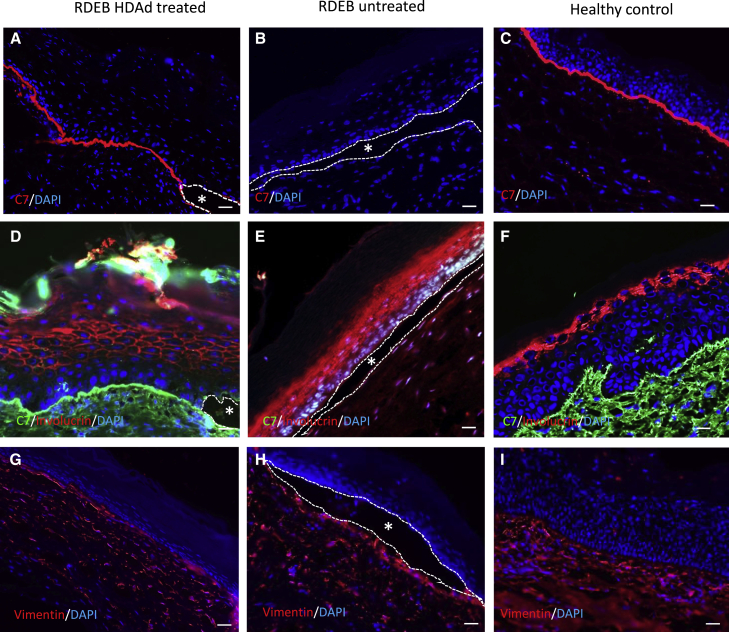
Long-term restoration of C7 in wounds treated with HDAd vector (A–C) Immunostaining with polyclonal anti-C7 antibody. (A) RDEB HDAd-treated skin. (B) RDEB untreated skin. (C) Healthy control skin. (D–F) Double staining with monoclonal anti-C7 antibody (green) and anti-human involucrin (red). (G–I) Immunostaining with anti-human vimentin. Cell nuclei stained with DAPI (blue). Asterisks denote blisters. Scale bar: 50 μm.

Immunostaining was performed with polyclonal (Figures 5A–5C) or monoclonal (Figures 5D–5F) anti-C7 antibodies. Almost continuous deposition of C7 was found along the dermal-epidermal junction in the re-epithelialized area after healing of the treated wound in patient grafts, comparable to staining along the BMZ detected in positive control tissue regenerated from normal human keratinocytes and fibroblasts (Figures 5C and 5F), although small blisters were found in regions showing less C7 deposition in the re-epithelialized area of treated grafts from patient cells (Figures 5A and 5D, asterisk). No C7 staining was found in regenerated untreated grafts from patient cells (Figures 5B and 5E). A double staining against human C7 and human involucrin was performed to show that the analyzed skin tissue had regenerated from human cells (Figures 5D–5F). The human origin of the dermis was demonstrated by staining with anti-human vimentin (Figures 5G–5I). Two independent long-term experiments were performed in which 3 individual mice carrying a graft derived from patient cells were treated with the vector in each experiment. Additional data from these experiments are shown in Figures S5A–S5E.

### Skin fragility test for evaluation of dermal-epidermal adhesion restoration

**Figure 6:**
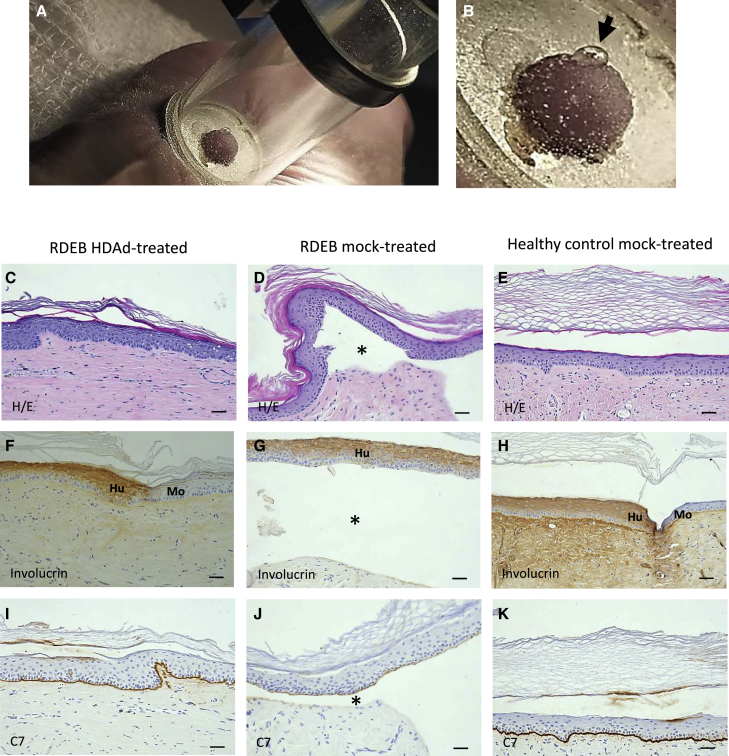
Skin fragility suction test (A and B) Blistering (black arrow) induced by suction in mock-treated RDEB re-epithelialized wound. (C–E) Histological analysis of grafts after suction test. (C) Non-detached, HDAd-treated RDEB re-epithelialized wound. (D) Dermal-epidermal detachment (asterisk) in mock-treated RDEB re-epithelialized wound. (E) Healthy control mock-treated re-epithelialized wound. (F–H) Anti-human involucrin staining showing human origin of healed tissue. (I–K) Immunohistochemical detection of C7. (I) Continuous C7 staining along the dermal-epidermal junction in the area after healing of the HDAd-treated wound. (J) Absence of C7 staining and dermal-epidermal detachment (asterisk) in mock-treated re-epithelialized wound. (K) Healthy donor mock-treated re-epithelialized wound, positive staining control. Scale bar: 50 μm.

To evaluate the long-term functionality of C7, 8 weeks after treatment with HDAd vector or mock treatment (fibrin gel only), we performed a suction stress test^9^ on healed areas. Mock-treated healed RDEB skin showed fragility as indicated by blistering (Figures 6A and B). In contrast, HDAd-treated RDEB skin showed similar resistance to healed skin on healthy grafts. Tissues were taken from the suctioned areas for characterization. Histological analysis showed grafts with normal skin architecture (Figures 6C–6E), with dermal-epidermal detachment only in the mock-treated RDEB skin (Figures 6), while adhesion was maintained in treated (Figure 6C) and healthy control skin (Figure 6E). Staining against human involucrin showed that the tissue in all grafts was of human origin and that epidermal differentiation was normal (Figures 6F–6H). Immunohistochemical detection of C7 showed continuous staining along the dermal-epidermal junction in the re-epithelialized area after healing of the treated wound (Figure 6I), comparable to staining in positive control tissue from healthy donor cells (Figure 6K). C7 was not detected in mock-treated healed grafts of the patient’s cells (Figure 6J).

### Repeated application of the vector to wounds of immunocompetent mice

The clinical scenario would require the repeated application of the Ad vector, both on a chronic wound several times and in the treatment of multiple wounds in different anatomical locations throughout the life of the patient. As a first approach to determine the viability of performing multiple treatments, we quantified neutralizing antibodies against Ad vectors of serotype 5 after one or two wound applications in immunocompetent C57BL/6 mice (Figure S6A). Antibodies were only detected after the second wound application, with a low titer compared with those arising after injection of the same dose of vector into the tail vein (Figure S6B). To test whether this immune response correlated with a decrease in vector infectivity, we analyzed whether wound-tissue transduction was reduced after the second vector application. Analysis of GFP expression in wound tissue harvested 3 days after vector delivery revealed comparable levels of tissue transduction after first and second treatments, showing that the antibodies generated after the first application to the skin are not sufficient to neutralize the vector (Figure S6C).

## Discussion

Although gene editing has made great strides in correcting disease in *ex vivo* strategies, where cells can be more easily transduced in cell culture followed by autologous transplantation, direct gene modification in tissues and organs would greatly extend therapeutic applications. We have now demonstrated that correction of the adhesion defect in the skin of patients with RDEB can be addressed by direct delivery of dual-guide CRISPR-Cas9 to the skin using Ad vectors. CRISPR-mediated editing of *COL7A1* resulted in C7 expression, and this correction was long lasting, since C7 was detected as a continuum along the dermal-epidermal junction 8 weeks after treatment with the Ad vector. Deletion of exons harboring pathogenic mutations by NHEJ-mediated gene editing strategies has been explored for the correction of inherited diseases associated with genes containing small in-frame exons encoding proteins with repetitive domains. Such is the case of the *DMD* gene encoding dystrophin in DMD and *COL7A1* encoding C7 in RDEB. Loss of the exon reframes the gene transcripts, resulting in the expression of an internally truncated, but partially functional, protein capable of correcting the disease phenotype. The feasibility of this NHEJ editing strategy has been demonstrated *ex vivo* and *in vivo* for DMD.^5^^,^^7^^,^^8^ Our laboratory has recently demonstrated the correction of the dermal-epidermal adhesion defect in RDEB skin by using an *ex vivo* strategy based on the electroporation of CRISPR-Cas9 RNPs in skin cells from patients with RDEB.^9^ Although a similar electroporation strategy has been tested in a mouse model of RDEB,^36^ its implementation in clinical practice seems unlikely given the impracticality of delivering high voltage electrical current to patients’ skin. The development of carriers, including viral vectors, for CRISPR delivery to skin *in vivo* was therefore warranted. *In vivo* delivery of CRISPR-Cas9 has been approached by using AAV and Ad vectors. For DMD, AAV vectors that can perform editing on significant fractions of muscle tissue have been shown to be useful in murine models of the disease.^5^^,^^7^ AAV vectors have also been used in an *in vivo* gene editing clinical trial recently conducted to correct congenital amaurosis by injecting these vectors directly into the subretinal space of the eye.^4^ Ad vectors expressing CRISPR-Cas9 have proven to be useful for *in vivo* gene editing in the liver of mice^23^ and for restoration of dystrophin expression in muscles in a mouse model of DMD^8^ using an exon excision strategy. β-Thalassemia correction has been recently reported in a murine model engrafted with human CD34+ thalassemic cells after *in vivo* transduction with a dual single guide RNA (sgRNA) CRISPR-Cas9-containing HDAd vector for restoration of fetal hemoglobin.^3^ While the performance of AAV vectors for skin delivery is very limited,^37^ Ad vectors have previously been successfully used to transduce human skin *in vivo* in a skin-humanized mouse model^38^ and in clinical trials to administer growth factors to non-healing wounds.^39^ We have now shown that HDAd vectors carrying CRISPR are suitable for skin transduction and specifically for gene-editing-mediated modification of the epithelial component of wounds, which allows considering their use for the treatment of the skin lesions characteristic of patients with RDEB. A limitation of our model is that it is based on the generation of acute wounds in regenerated skin of patients in immunodeficient mice. Chronic wounds that often progress to SCC are certainly one of the key problems in RDEB. Modeling the complexity of chronic wounds involves reproducing the inflammatory and fibrotic component of the wound environment and poses a challenge that we did not address in this study.

Significant hurdles for *in vivo* delivery of CRISPR-Cas9 into intact tissues still remain and will have to be addressed in future preclinical work. The immune reaction against Ad vector proteins could decrease the effectiveness of the treatment, especially if sequential applications are needed, and therefore potentially poses a significant obstacle for *in vivo* therapy. However, here we have demonstrated a successful transduction of wound tissue 1 month after the first treatment with our HDAd-CRISPR-Cas9 vector in immunocompetent mice, suggesting that the immune response elicited does not cause vector neutralization in this protocol. In addition, unlike other gene therapy approaches that will probably require sustained vector expression,^21^ modifications introduced by gene editing into epidermal stem cells are potentially long lasting, and therefore the limited duration of Ad vector expression might not be an impediment to therapeutic use. In any case, high preexisting adaptive immunity against Ad5 could be addressed by the vectorization of alternative serotypes.^40^^,^^41^ Future strategies combining immuno-evasive herpes vectors with CRISPR tools to achieve long-lasting modifications in *in vivo* therapy of RDEB lesions are also conceivable.

Another matter of concern for *in vivo* implementation of CRISPR-Cas9 gene editing is the potential preexisting immunity against Cas9 proteins, since the widely used Cas9 orthologs from *Streptococcus pyogenes* and *S. aureus* originate in human commensal bacteria.^42^^,^^43^ Although genome editing was not impeded in the liver of mice with preexisting immunity to SaCas9, a cytotoxic T cell response caused elimination of genome-edited cells followed by liver regeneration.^44^ Whether this cytotoxic reaction happens in the skin of immunocompetent mice treated with HDAd vectors for SaCas9 expression will be studied in the future. However, the potential limitations to *in vivo* therapeutic use of CRISPR-Cas9 due to adaptive immune responses to Cas9 proteins could be overcome by using Cas9 orthologs from microorganisms that do not reside in humans or by transient immune suppression during the course of treatment to prevent severe immune responses.

We have demonstrated that Ad vectors can be used to deliver the CRISPR-Cas9 system to human RDEB skin in a humanized mouse model to address the correction of the dermal-epidermal adhesion defect by *in vivo* gene editing. This achievement paves the way for therapeutic protocols based on *in vivo* gene editing for direct treatment of lesions in patients with RDEB. Topical administration of gene-correction vectors would allow the treatment of chronic open wounds that end up causing extensive tissue damage and deteriorating the patient’s quality of life, as well as the treatment of internal mucosal areas that are not susceptible to skin grafting procedures, which could represent an important advance in the management of RDEB.

## Materials and methods

### Ad vectors construction

An all-in-one construct for expression of SaCas9 and gRNAs targeted at sequences flanking *COL7A1* exon 80 was initially constructed using plasmid pX601-AAV-CMV:NLS-SaCas9-NLS-3xHA-bGHpA; U6:BsaI-sgRNA (Addgene #61591). The following oligonucleotides were annealed, phosphorylated with T4 PNK, and ligated into the BsaI site of pX601 to produce pX601-derived plasmids: pX601-sg1: 5′-CACCTCATCAGTGCCCTCTCTATG-3′ and 5′-AAACCATAGAGAGGGCACTGATGA-3′; pX601-sg2: 5′-CACCGGCAAGACAGGTGAAGGTTC-3′ and 5′-AAACGAACCTTCACCTGTCTTGCC-3′. An Asp718/NotI fragment from plasmid pX601-sg1 containing the U6 promoter and gRNA was cloned into the Asp718 site of pX601-sg2 after blunting with Klenow enzyme to generate a dual gRNA pX601-derived construct. The CMV promoter in this plasmid was replaced by the PGK promoter by ligating an EcoRI/XmaI fragment from PGK-GFP plasmid into XbaI/AgeI sites of pX601-dual guide after blunting EcoRI and XbaI by Klenow filling to generate PGK-601-dg plasmid. An EcoRI fragment containing the PGK-Cas9 cassette from the resulting plasmid was cloned into PGK-TAL plasmid. A SnaBI/NotI fragment from the dual gRNA pX601 construct containing both U6-gRNA cassettes was cloned into the EcoRV site from the previous plasmid in a 5′ position from the PGK-Cas9 cassette to generate the dgPGKSaCas9 plasmid. A NheI/EcoRI fragment from this plasmid was cloned into pDC315iGFP to generate a shuttle plasmid for FGAd vector generation, designated as pDC315-dg-PGK-Cas9iGFP.^26^ For generation of the HDAd vector, an EcoRI/NotI fragment containing the IRES-GFP sequence from pLZRiGFP plasmid was cloned into the AflII site of dgPGKSaCas9 after blunting with Klenow, and a KpnI site was replaced by BssHII by inserting a double-stranded oligonucleotide. A BssHII/NotI fragment containing expression cassettes for both gRNAs and Cas9IRESGFP was then cloned into AscI/NotI sites of pC4HSU to generate the 37 kb vector plasmid.

### Generation of viral vector particles

For FGAd vector production, the pDC315-dg-PGK-Cas9iGFP shuttle plasmid was co-transfected with pBHGloxΔE1, 3Cre (Microbix Biosystems, Mississauga, ON, Canada) into HEK293 cells using calcium phosphate.^26^ One day after transfection, the cells were overlayed with a 1% agarose gel containing 0.2% yeast extract. Lysis plaques were isolated after 15–20 days, and viral particles were used to infect HEK293 cells from which viral genome DNA was isolated and analyzed by restriction analysis with EcoRI. Vector preparations with the correct pattern were expanded on HEK293 cells and purified by Cesium density gradient centrifugation.

For HDAd viral particles, the HD vector plasmid was transfected into HEK293Cre cells, and cells were then infected with the FG helper virus.^28^ To increase the titer of the viral vector particles, serial passages involving sequential coinfections of HEK293Cre cells with helper virus and viral vector particles generated in the previous round were made. In each passage, once the cytopathic effect was observed, the cells together with the supernatant were collected and frozen at −70°C to carry out the subsequent amplification rounds. To purify and concentrate the final amplification of the viral supernatant, a standard CsCl gradient protocol was used.^45^ The titer of the vector preparations was determined as viral genomes (vg)/mL as described.^46^

### Primary cultures of human keratinocytes and fibroblasts

Human dermal keratinocytes and fibroblasts were isolated from skin biopsies obtained from both healthy donors and patients who were homozygous carriers of the c.6527dupC mutation in the *COL7A1* gene.^47^ Skin biopsies were obtained from patients after approval from the ethics committee of the collaborating hospital upon informed consent. Primary human RDEB and healthy donor keratinocytes and fibroblasts were cultured as previously described.^48^ Cells were cultured at 37°C in a humid atmosphere containing 5% CO_2_. The culture medium was changed every 2 days.

### Cytotoxicity analysis by cytometry

To assess the viability of patient keratinocytes after transduction with FGAd and HDAd, cells were transduced with both types of Ad vectors at different MOIs, and DAPI permeability was analyzed by flow cytometry in an LSR Fortessa (BD) cytometer.

### Genotyping of gene-edited keratinocytes and fibroblasts

Genomic DNA was isolated from keratinocytes and fibroblast lysates by isopropanol precipitation (lysis buffer was Tris [pH 8] 100 mM, EDTA 5 mM, SDS 0.2%, NaCl 200 mM, and 1 mg/mL proteinase K [Roche Diagnostics, Mannheim, Germany]) and resuspended in Tris/EDTA (TE) buffer. Approximately 20–50 ng genomic DNA was used for PCR amplification. PCR fragments spanning the nuclease target sites were generated with primers F1/R (F1, 5′-gtgagtggtggctgaagcac-3′; and R, 5′-accccaccaaggaaactga-3′). PCR program TD 68-63 was as follows: 94°C for 5 min; 5 cycles of 94°C for 30 s, 68°C for 30 s, and 72°C for 30 s, decreasing annealing temperature 1°C every cycle; followed by 30 cycles of 94°C for 30 s, 63°C for 30 s, and 72°C for 30 s; then 72°C for 7 min. PCR products were analyzed in 1.5% agarose gel. Molecular weight marker was IX (Sigma-Aldrich) and 100 bp ladder (Biotools).

### *In vivo* testing on humanized skin mouse model

Animal studies were approved by our institutional animal care and use committee according to national and European legal regulations. RDEB or healthy keratinocytes were seeded on fibrin dermal equivalents containing RDEB or healthy fibroblasts, respectively, prepared as previously described.^33^ Bioengineered skin equivalents were grafted onto the back of 7-week-old female immunodeficient mice (nu/nu, NMRI background, Elevage-Janvier, Le Genest-Saint-Isle, France), as previously described.^49^ Grafting was performed under sterile conditions, and mice were housed in pathogen-free conditions for the duration of the experiment at the CIEMAT Laboratory Animals Facility (Spanish registration 28079-21 A). All handling was carried out under sterile conditions, and all experimental procedures were according to European and Spanish laws and regulations. Grafts were monitored using a magnifying lens equipped with white light.

At 12 weeks after transplantation, engrafted human skin was injured with 2 mm biopsy punches (Stiefel Lab), and the HDAd vector was delivered embedded in fibrin.^38^ Briefly, viral particles (1010 vg) were added to 30 μL fibrinogen (from blood cryoprecipitates) and 6.6 μL human thrombin (Sigma, St. Louis, MO, USA) diluted in 0.025 mM CaCl_2_ (Sigma). The mixture was applied into the wound and allowed to clot. Mice were then sacrificed at different time points post-treatment, and grafts were harvested for immunohistochemistry analyses.

### Quantification of neutralizing antibodies against human adenovirus type 5

Excisional wounds were made in the skin of the back of immunocompetent adult C57BL/6 mice using a 2 mm punch, and the HDAd vector (1010 vg) was delivered into the wounds embedded in fibrin as described above. For systemic administration, the same dose was injected through the tail vein. Blood serum was collected at different times after treatment and stored at −20°C until use. A FGAd encoding luciferase (Vector Biolabs, Philadelphia, PA, USA) was incubated for 1 h with serum dilutions, and then the mixture was added to A549 cells in a 96-well plate. Infection was maintained for 24 h under standard cell culture conditions, and then luciferase activity was quantified in cell lysates. The neutralizing antibody titer corresponds to the dilution showing 90% luciferase activity inhibition (IC_90_).

### Immunofluorescence and immunohistochemical staining

Skin biopsies were embedded in OCT (Optimal Cutting Temperature) compound , frozen, and cut at 5 μm cryosections. After being fixed by exposure to air, sections were blocked in PBS with 3% BSA (Sigma) for 30 min and were then analyzed using specific primary antibodies against GFP (Invitrogen), human Involucrin (SY5 monoclonal antibody, Sigma), 2 different antibodies against C7: monospecific polyclonal anti-C7 N terminus antibody^34^ (a gift from Dr. Nystrom, University of Freiburg, Germany) and monoclonal anti-human C7 (clone LH7.2, Sigma) and human cytokeratin 14 (sc-23878, Santa Cruz Biotechnology). Secondary antibody Alexa Fluor 488 (Invitrogen) was used at 1:1,000 dilution. Preparations were then mounted using Mowiol (Hoechst, Somerville, NJ, USA) mounting medium and DAPI 20 mg/mL (Sigma) for nuclei visualization.

Formalin-fixed paraffin sections (4–6 μm) were stained with hematoxylin and eosin (Gill 2 hematoxylin and eosin Y, alcoholic; Thermo Fisher Scientific, Altrincham, UK) following a standard procedure to determine tissue architecture. Immunoperoxidase detection of C7 in paraffin-embedded, formalin-fixed sections was carried out with proteinase K antigen retrieval. Immunoperoxidase staining for human involucrin was performed using rabbit SY5 monoclonal antibody (Sigma) on paraffin sections without antigen retrieval. The ABC peroxidase kit (Vector Laboratories) with diaminobenzidine as a substrate was used.

### WB analysis

Confluent fibroblast and keratinocytes cultures were lysed in buffer containing 1% Nonidet P-40, 25 mM Tris-HCl (pH 7.4), 100 mM NaCl, and protease and phosphatase inhibitor cocktails (Roche). Protein extracts were electrophoresed on NuPAGE Novex 3%–8% Tris-Acetate gel electrophoresis (Invitrogen, Carlsbad, CA, USA) and electrotransferred to nitrocellulose membranes (Invitrogen). Membranes were blocked with 5% non-fat milk powder in TBS for 1 h at room temperature (RT) and incubated overnight with a monospecific polyclonal anti-C7 antibody^34^ (a gift from A. Nystrom, University of Freiburg, Germany) and vinculin (ab130007, Abcam). Detection was performed using HRP-conjugated secondary antibodies and a chemiluminescent detection assay.

### Data availability

The authors confirm that the data supporting the findings of this study are available within the article and its supplemental information. Additional sequencing data are available upon request.

## Supplemental information

Document S1. Figures S1–S6

Document S2. Article plus supplemental information
