# Supplementary material for: Preclinical model for phenotypic correction of dystrophic epidermolysis bullosa by in vivo CRISPR-Cas9 delivery using adenoviral vectors
Source: Mol Ther Methods Clin Dev. 2022 Sep 16;27:96–108. doi: 10.1016/j.omtm.2022.09.005 (PMC9531050; doi:10.1016/j.omtm.2022.09.005)
Supplement: Document S1. Figures S1–S6 [file mmc1.pdf]

OMTM, Volume *27*

Supplemental information

Preclinical model for phenotypic correction of dystrophic epidermolysis bullosa by *in vivo* CRISPR-Cas9 delivery using adenoviral vectors

Marta García, Jose Bonafont, Jesús Martínez-Palacios, Rudan Xu, Giandomenico Turchiano, Stina Svensson, Adrian J. Thrasher, Fernando Larcher, Marcela Del Rio, Rubén Hernández-Alcoceba, Marina I. Garín, Ángeles Mencía, and Rodolfo Murillas

Figure S1

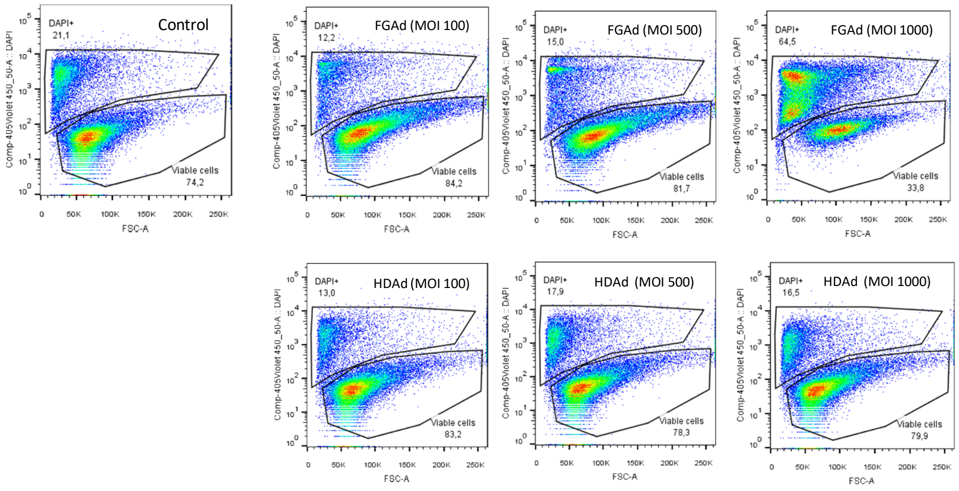

Figure S1.Viability of treated cells. DAPI permeability analysis of viability of patient keratinocytes after transduction with FG and HDAd vectors. Cells were transduced with FGAd or HDAd vectors at 100, 500 and 1000 multiplicities of infection (MOI) and DAPI permeability was analyzed by flow cytometry. Control, untransduced patient keratinocytes.

Figure S2

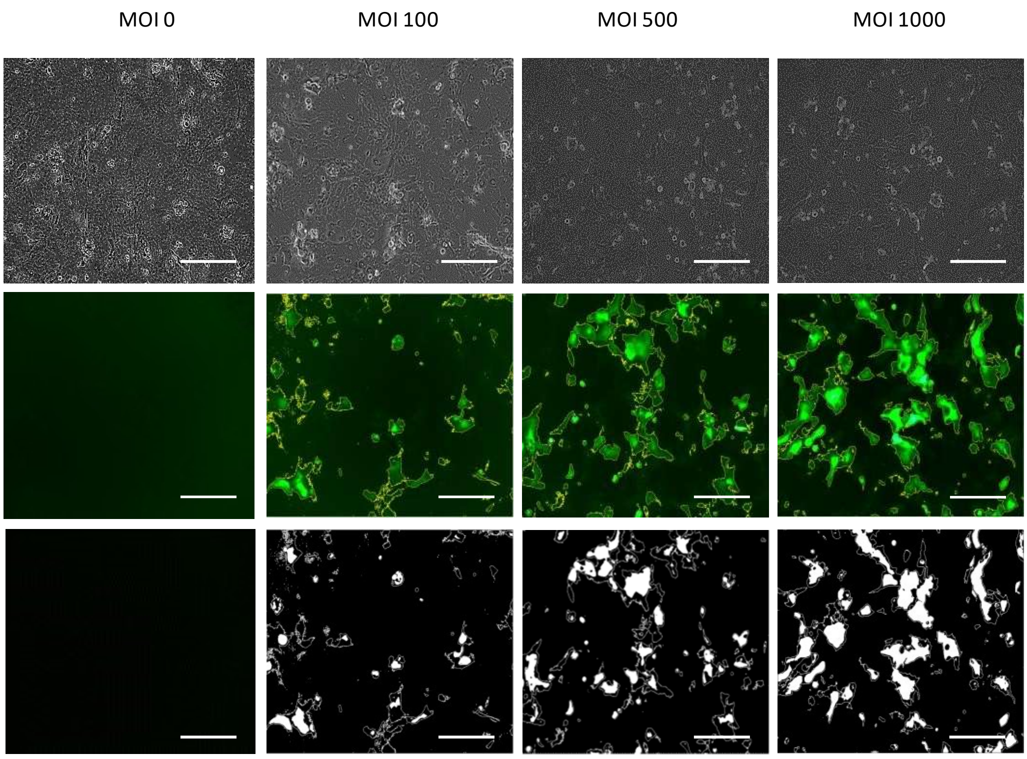

	MOI 0	MOI 100	MOI 500	MOI 1000
% of Fluorescent area	0,0	24,23	32,166	43,197

Figure S2. Evaluation of in vitro transduction by quantification of GFP fluorescent areas. Segmented images are binary images. The background (black) is labeled with the lowest pixel value while the cells (white) are labeled with the height pixel value. Cellular confluence was 100%.The calculation of the infection ratio was based on the percentage of cell fluorescence area with the ImageJ program. Scale bar: 100 µm.

Figure S3

C7

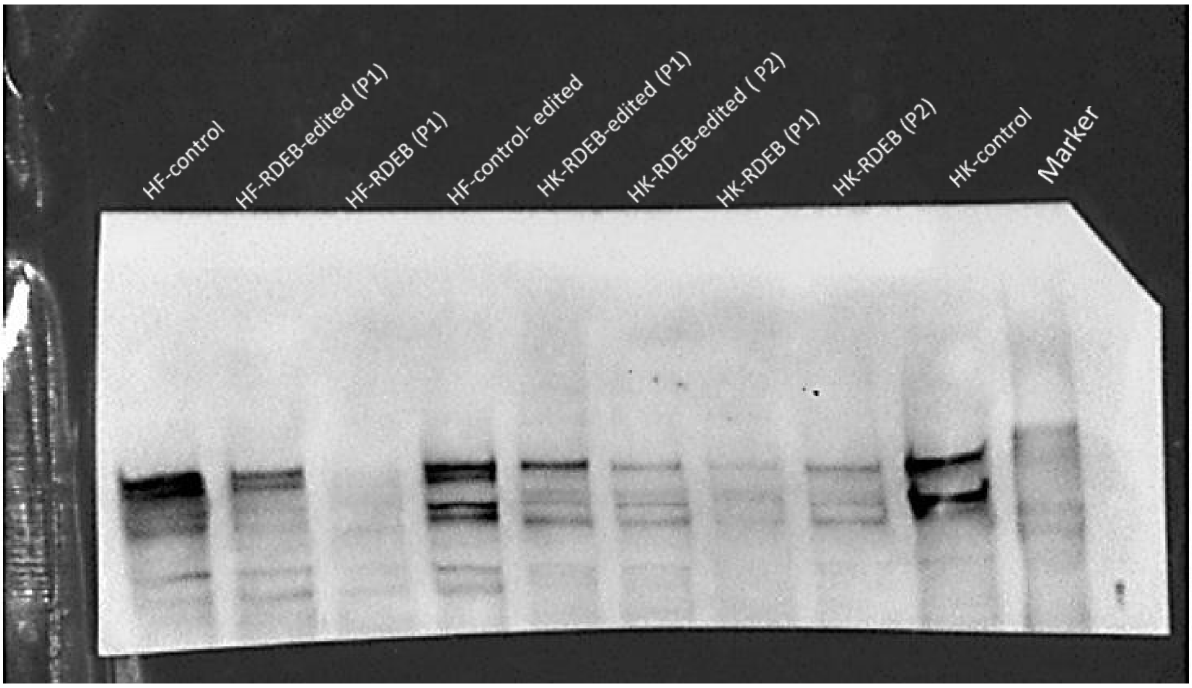

Figure S3. Western blot analysis of C7 expression in patient cells. Original membrane for WB data shown in Fig3. HF, human fibroblasts. HK, human keratinocytes. RDEB, patient cells. Control, healthy donor cells. Cells (HFs and HKs) from two patients (P1 and P2) were treated with HDAd vector (edited).

Figure S4

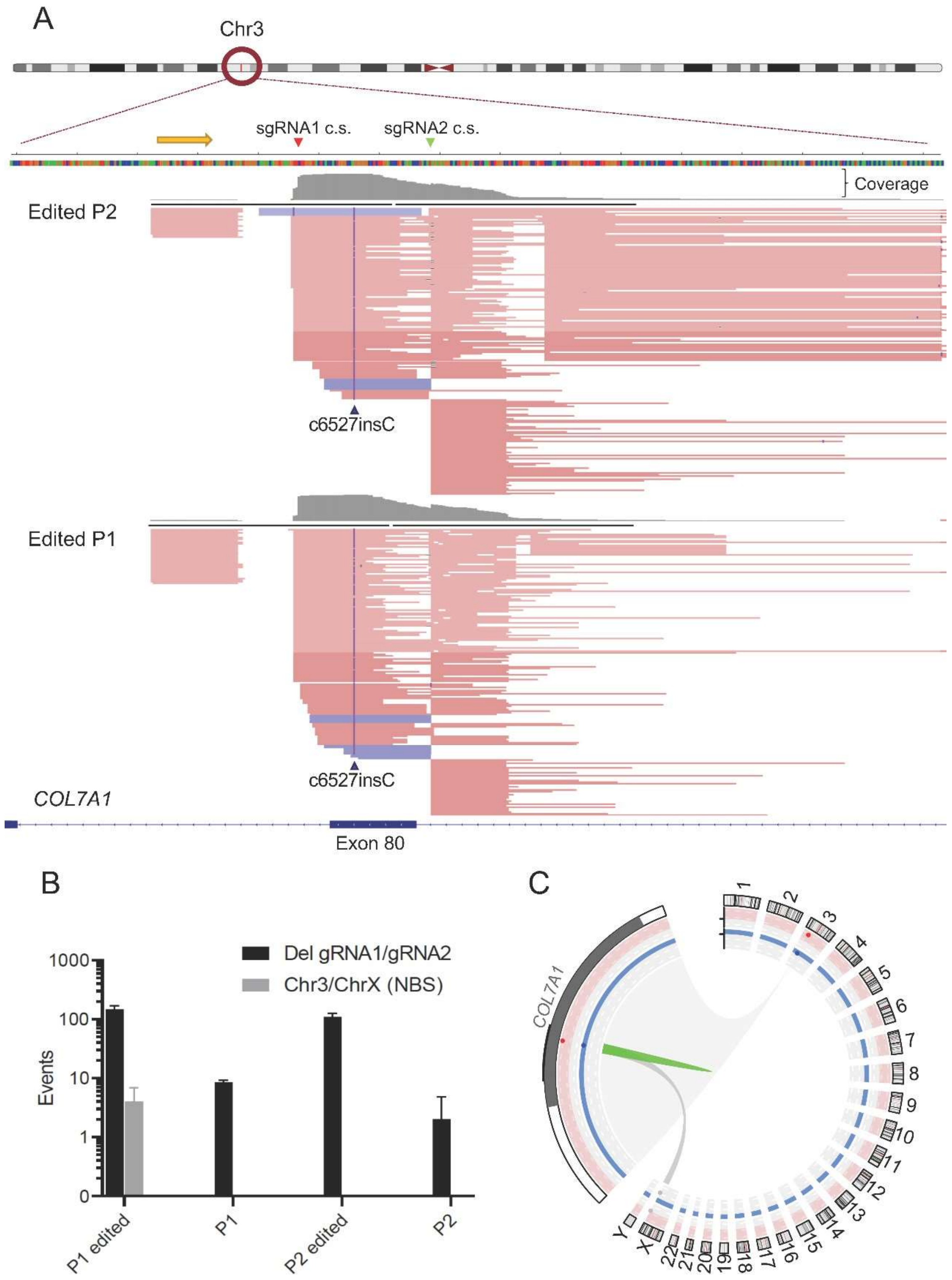

Figure S4. CAST-seq based-detection of chromosomal translocation in gene edited RDEB primary keratinocytes.(A) Qualitative CAST-Seq analysis. Integrative Genomics Viewer (IGV) plots illustrate CAST-Seq reads surrounding the target site within a window of 350 bp. Mapped CAST-Seq reads are represented by bars. Purple and red bars indicate sequences aligning with the negative or positive strand, respectively. Cutting sites of both guides are indicated (sgRNA1 c.s. and sgRNA2 c.s.). *COL7A1* nested bait primer is indicated with a yellow arrow. (B) Quantitative analysis of different chromosomal rearrangement events found in P1 and P2 edited samples versus P1 and P2 (untreated samples). Del gRNA1/gRNA2 represents events containing the deletion generated by the two CRISPR-Cas9 guides. (C) Circos plot representing the chromosomal aberration retrieved by CAST-seq. *COL7A1* target region showed on the left. On-target site cluster is shown in green. Significant scores are pointed up by red dots (on-target-mediated translocations) or blue dots (homology-mediated translocation). Gray dots represent Natural double-strand-Break Site, NBS.

Figure S5

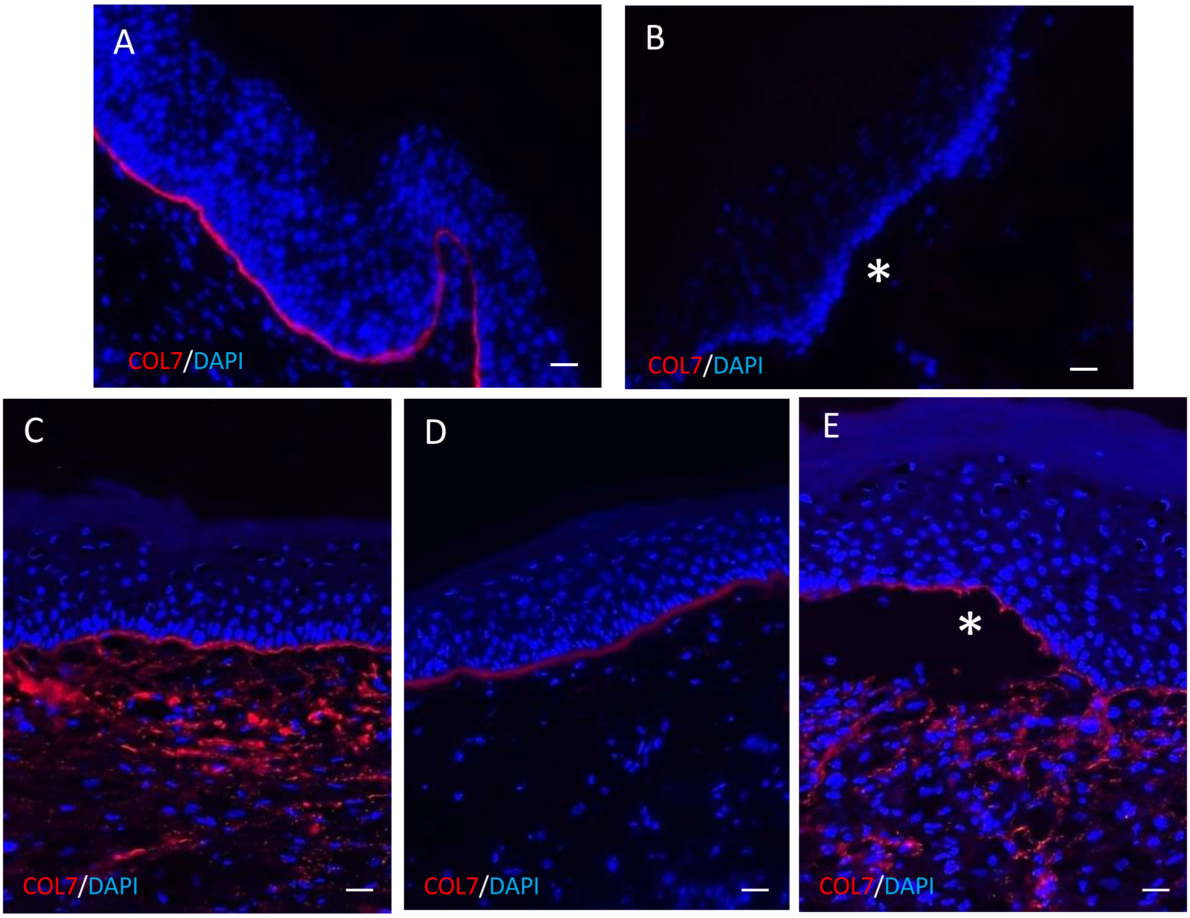

Figure S5. Long term analysis of C7 in wounded areas eight weeks after treatment with HDAd vector. Data from three grafts treated in two independent experiments are shown. IF with a polyclonal anti-C7 antibody (red). A) Healthy donor graft, positive control; B) Untreated patient graft, negative control; C-E) Treated patient grafts. Cell nuclei stained with DAPI (blue). Asterisks denote blisters, in untreated negative control (B) and in a region with less C7 deposition in a treated graft (E) . Scale bar 50µm.

Figure S6

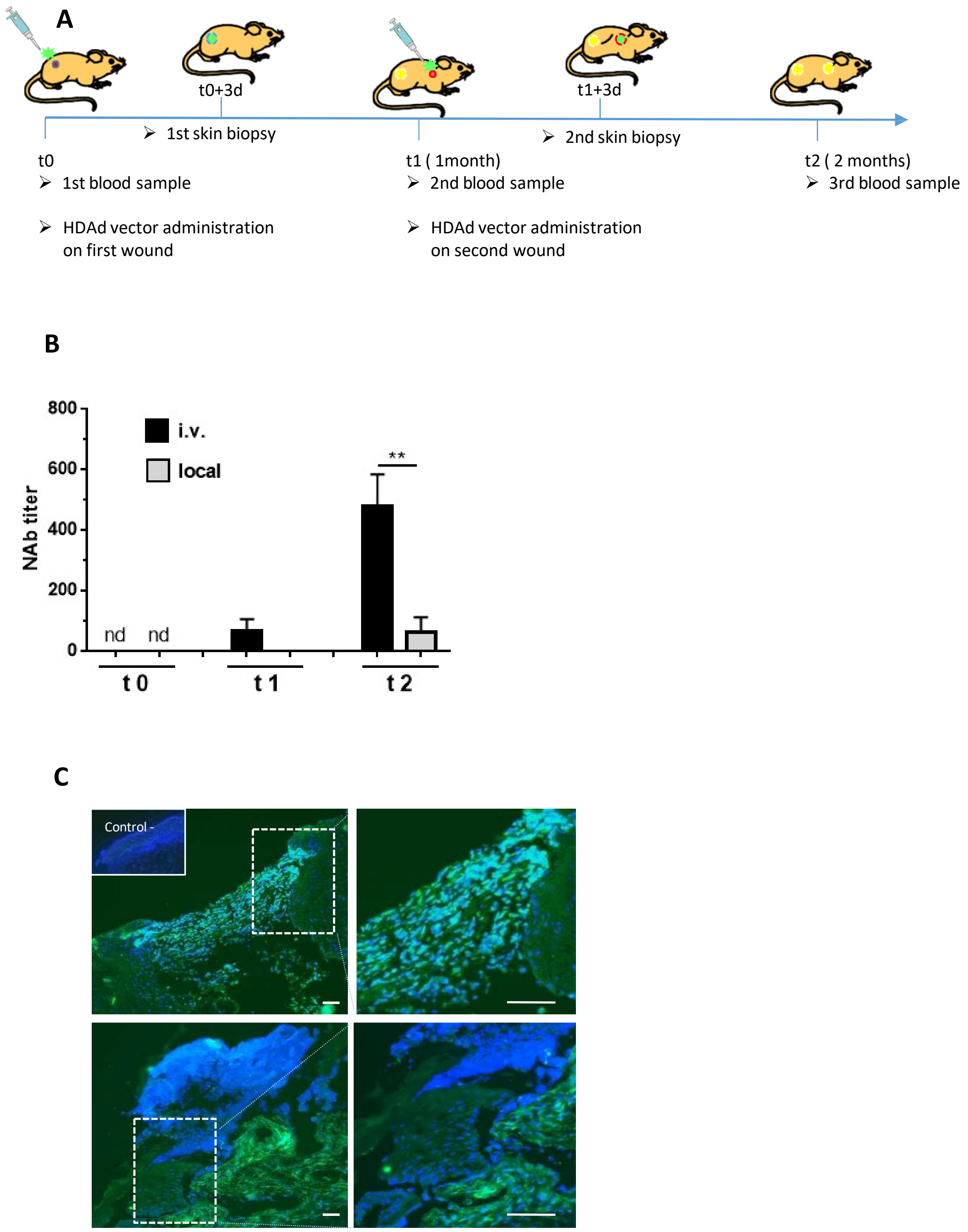

Figure S6. Quantification of neutralizing antibodies against AdV serotype 5 after repeated administration of AdV vector. A) Schematic showing the experimental design. B) quantification of neutralizing antibodies against human adenovirus type 5 in mouse blood, serum was collected at several time points (t0, t1, t2) after treatments as shown in A. The neutralizing antibody titer corresponds to the dilution showing 90% luciferase activity inhibition (IC_{90}). The graph shows the mean of two independent experiments n = 6 for each administration method (p=0.0087, Mann Whitney U test. C) Analysis of GFP expression in wound tissue harvested three days after vector delivery. Upper panels, 1st skin biopsy (t0+3d). Inset, negative control (untreated). Lower panels, 2nd skin biopsy (t1+3d). Right panels, higher magnification of the boxes in left panels. Scale bar: 50µm.
